# Population of Northern Portugal: Study of Genetic Diversity and Forensic Parameters of 26 Y-STR Markers

**DOI:** 10.3390/genes17010101

**Published:** 2026-01-19

**Authors:** Bárbara Maia, Jennifer Fadoni, Laura Cainé, Luís Souto, António Amorim

**Affiliations:** 1 Biology Department, University of Aveiro, 3810-193 Aveiro, Portugal; barbaramaia06@ua.pt (B.M.);; 2 Nacional Institute of Legal Medicine and Forensic Sciences, I.P., North Branch, 4050-202 Porto, Portugal; jennifer.n.fadoni@inmlcf.mj.pt; 3 Faculty of Medicine, Lisbon University, 1649-028 Lisboa, Portugal; 4 Nacional Institute of Legal Medicine and Forensic Sciences, I.P., Center Branch, 3000-548 Coimbra, Portugal; laura.m.caine@inmlcf.mj.pt; 5 Faculty of Sciences, Lisbon University, 1749-016 Lisboa, Portugal; 6 LAQV&REQUIMTE, Applied Chemistry Laboratory, Department of Chemical Sciences, Faculty of Pharmacy, Porto University, 4050-313 Porto, Portugal

**Keywords:** Northern Portugal, Y-STR, Y-chromosome, haplogroups, population genetics, forensic genetics, genetic diversity

## Abstract

**Background**: Short tandem repeats (STRs) are highly variable sequences present along the human genome, including the Y-chromosome. Y-STRs are exclusive to males, and the haplotypes they define are informative. **Objectives**: Twenty-six Y-STR loci were genotyped in 252 males from Northern Portugal to characterise Y-chromosome genetic variation using the Investigator Argus Y28 QS Kit. **Methods**: The kit mentioned was used to amplify male DNA samples, and capillary electrophoresis was used to analyze the fragments. Forensic parameters and haplotype diversity were computed, and samples’ haplogroups were predicted. A multidimensional scaling (MDS) plot was used to graphically represent the **R**_ST_ genetic distances, including reference populations. **Results**: A total of 250 different haplotypes were observed, including 248 unique ones, yielding a very high haplotype diversity (HD = 0.999) and discriminatory power (DP = 0.992). Haplogroup analysis indicated a predominance of R1b (58.7%), followed by E1b1b, I and J, pointing to a population history shaped by Mediterranean and North African gene flow. Comparative analysis between Portugal and 5 other populations showed greater genetic affinity with Spain and Italy, while revealing marked differentiation from Greece, Morocco, and former Portuguese colonies. **Conclusions**: The results confirm that the Northern Portuguese Population exhibits high Y-STR variability and robust forensic resolution. The dataset was submitted to the YHRD database, enhancing the representation of the Portuguese population and underscoring the value of the 26 locus panel for applications in forensic science, genealogy, and population genetics.

## 1. Introduction

Portugal is a country on the Iberian Peninsula that borders Spain in Europe’s Southernmost part. It is known for once having a global empire, built from the power gained from the Maritime Discoveries. Numerous civilisations, including Celts, Moors, Visigoths, and Romans, have occupied Portugal, and the region has a history of repeated invasions that have moulded the current population [1,2]. During the Roman invasion of Portugal, extensive mineral exploitation and Mediterranean trade drove economic growth, the construction of infrastructure, and urbanisation. Roman culture, law, and the Latin language spread throughout Western Iberia, gradually assimilating native populations as individuals from other regions of the Empire, including Europe, North Africa, and the Eastern Mediterranean, came [3]. After the Algarve was conquered by 1249, the Kingdom of Portugal expanded and solidified its borders in the late Middle Ages [4].

**Figure 1:**
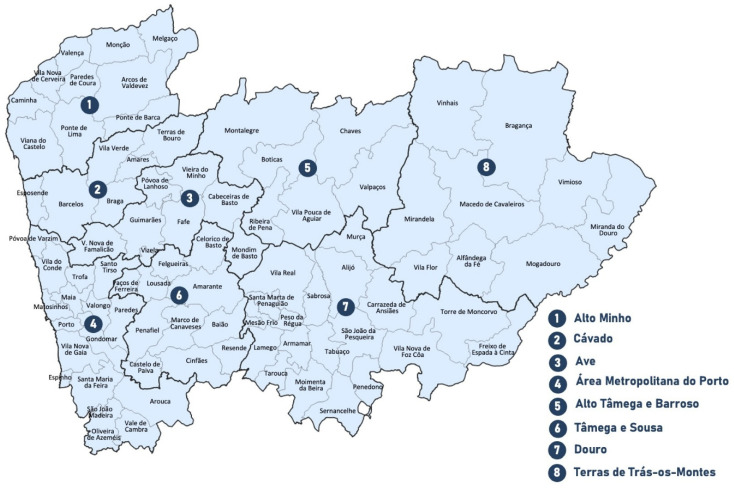
Official borders of the Northern Region of Portugal (Adapted from https://www.ccdr-n.pt/pagina/regiao-norte/apresentacao, accessed on 6 March 2025).

The Northern region of Portugal consists of a total of 86 municipalities, and the population as we know it today was shaped by several successive aforementioned invasions [1]. The Northern region and it’s municipalities are represented in Figure 1.

Previous Y-STR studies of Portuguese populations were conducted with early-generation 9- to 17-locus panels and did not include RM-STRs [5,6,7]. No high-resolution 26-STR dataset representing Northern Portugal is currently available in YHRD, as no recent population studies have been conducted focusing on the population of northern Portugal; the present study is essential for understanding its genetic composition and possibly elucidating all of the emigration and immigration that gave rise to the current population. In addition, it can also help to better understand certain events of the past, such as the invasions of all of the different civilisations that contributed to the genetic diversity of said population, as the Y-chromosome is a powerful tool for studying human populations and evolutionary pathways. This is due to the fact that the non-recombining part of the Y keeps track of all of the mutations that have happened in male lineages over the course of evolution [8]. The lack of representability of Portuguese populations in Y-STR databases can also be addressed by this study, because, much like Sanchéz-Méndez et al., [9], our population lacks in representability, which can be a challenge in forensic casework such as sexual crimes or individual identification.

Given its paternal inheritance, the Y-chromosome is shared among males of the same paternal lineage, who generally exhibit highly similar Y-STR haplotypes. However, this transmission is not strictly identical across generations, as the Y-chromosome is subject to not only mutational events but also other mechanisms such as recombination within the pseudoautosomal regions (PARs) and gene conversion between palindrome arms. These processes contribute to sequence variation and should be considered when interpreting paternal lineage relationships. These aspects make them suitable for kinship studies, human identification, and even historical genetic genealogy when there is no direct male descendant [10,11]. Short tandem repeats (STRs) are highly variable sequences present along the human genome, including the Y-chromosome, and consist of repeated copies of a 1–6 bp motif [12]. Y-STRs are a common tool in forensic investigations, as they are exclusive to males, and the haplotypes they define are informative [13].

The primary goals of this study are to perform Y-chromosome DNA typing on individuals from a sample of the Northern Portuguese population, to identify all of the haplotypes, and to use Y-STR analysis to predict the related haplogroups. Lastly, the results will be added to the YHRD database, which will increase the overall number of Portuguese Haplotypes in the database and further diversify the data present [14].

## 2. Materials and Methods

### 2.1. Sample Collection

A total of 252 samples were analysed, including 21 buccal swabs and 231 blood stains, all from male individuals residing in the Northern Region of Portugal, whose parents were born in the same region, as provided by information obtained from mandatory case documentation to ensure paternal ancestry from the Northern region. The samples were collected during paternity investigations carried out at the National Institute of Legal Medicine and Forensic Sciences, Portugal (INMLCF). Factors like age distribution or jobs were not accounted for, as they present no relevance to this study.

This study was approved by the Ethics Committee of the Institute of Legal Medicine and Forensic Sciences, while also being approved by the Department of Information, Training, and Documentation of the Institute (Acceptance number: CE-44/2024). The use of these samples complies with the Portuguese legislation (Decree-Law No. 45/2004 of 19 August; Decree-Law No. 12/2005, of 26 January; Decree-Law No. 849/2010), as well as with the National Institute of Legal Medicine and Forensic Sciences’ regulations, in which it is permitted to use samples stored for more than 2 years after the caseworks are completed, as long as fully anonymized. All samples belonged to cases legally closed and from archived forensic caseworks dating from 1 January 2021, to 20 January 2023. Although such samples do not constitute a randomised population survey, this approach is ethically compatible with the retrospective use of anonymised material. To prevent clustering, only one sample per independent case was included.

### 2.2. DNA Extraction and Analysis

The 21 samples that were buccal swabs were submitted to DNA extraction before the amplification process. Two separate extractions were performed, both automated, using the PrepFiller Express Forensic DNA Extraction Kit (Qiagen, Hilden, Germany) and the AutoMate Express Forensic DNA Extraction System (Applied Biosystems, Waltham, MA, USA). The same conditions and the manufacturer’s instructions [15] were followed for both. The extracted samples were kept at −20 °C until they were amplified following the completion of the extractions.

All blood stains were amplified directly according to the manufacturer’s protocol [16], with the following alterations: the reaction volume was reduced by half, from 25 µL to 12.5 µL, and the number of cycles was reduced to 28, from 30, to facilitate reagent optimisation and prevent overamplification. These alterations were based on an internal validation performed at the National Institute of Legal Medicine and Forensic Sciences, North Branch. Internal validation included sensitivity testing, peak height balance assessment, and replicate concordance analysis. Complete profiles were consistently obtained from DNA inputs, with no systematic allelic drop-out and balanced heterozygous peak height ratios across all loci. Replicate amplifications showed full concordance, supporting the reliability of the modified protocol for forensic and population genetic applications. The kit used in this study was the Investigator Argus Y28 QS (Qiagen, Hilden, Germany), and the STRs analysed were as follows: DYS389-I, DYS391, DYS389-II, DYS533, DYS390, DYS485, DYS393, DYS19, DYS437, DYS460, YGATAH4, DYS448, DYS439, DYS549, DYS438, DYS456, DYS643, DYS635, DYS385 and DYS392, DYS449, DYS481, DYS570, DYS576, DYS518 and DYS627 of which the last 6 STRs are Rapid Mutation STRs (RM-STRs), with DYS3895 being the only multicopy locus.

The cycling protocol used was as follows: pre-denaturing was carried out at 96 °C for 12 min, followed by the denaturing process of 10 s at 96 °C, annealing started with lowering the temperature to 61.5 °C for 1 min and 25 s, and the extension process, which lasts 5 s, had the temperature raised to 72 °C. These three steps are repeated for 28 cycles. This is followed by a final extension, which begins with a 5-min process at 68 °C, followed by a second process with the same duration but at a temperature of 60 °C, with maintenance at 10 °C.

A second amplification, with the goal of obtaining an identifiable genetic profile, was carried out with all samples that did not have satisfactory results, such as possible mutations present, or samples that presented little or no DNA, preventing haplotype determination.

Capillary electrophoresis was used to genotype the PCR results using Applied Biosystems 3500 Genetic Analyser (Applied Biosystems, MA, USA) in accordance with the manufacturer’s instructions. GeneMapper (version 1.6) (Applied Biosystems, MA, USA) was used for genotyping, and allele designations were based on comparisons with the allelic ladder included in the kit.

### 2.3. Statistical Analysis

Using a direct count method, Office Excel v2508 was utilized to calculate the number of distinct haplotypes, the frequency of unique haplotypes (FUH), discriminatory capacity (DC), match probability (MP) and haplotype diversity (HD). To calculate the haplotype diversity, the formula HD = n(1 − Σpi^2^)/(n − 1) was used. In the current formula, pi is the frequency of the i-th haplotype, and n is always the same size. The MP, MP = Σpi^2^, where pi is the frequency of the i-th haplotype, was calculated through the addition of the squared relative frequencies of each observed haplotype. The DC was calculated as the ratio of distinct haplotypes to total individuals, and the UHF was calculated as the ratio of unique haplotypes to total individuals [17].

The forensic parameters, such as allele frequencies at each locus, gene diversity (GD), polymorphism information content (PIC), and power of discrimination (PD), were calculated using STRAF Software (STR Analysis for Forensics) version 2.2.2 [18]. The R_ST_ and R_ST_ *p*-values between populations were calculated using Arlequin Software 3.5.2.2 [19], and a Multidimensional Scaling plot (MDS) was computed using R Software 4.5.1 [20] to visualize the relationships between the populations used, based on the calculated matrix of pairwise genetic distances.

## 3. Results

### 3.1. Haplotypes

A total of 252 haplotypes were obtained from the 252 Northern Portuguese samples. Two individuals shared one of the haplotypes discovered, whereas the other two people shared another haplotype; therefore, one sample from each pair was removed from the analysis. This resulted in 250 distinct haplotypes, 248 of which were unique, meaning that the unique haplotype frequency was 0.984. The results showed that the DC was 0.992 and the HD was 0.999. Complete haplotype data for all 26 Y-STR loci is shown in Table S1 and is in the process of being inserted into the YHRD database.

The distribution of allele frequencies for the 26-STR loci is shown in Figure S1, and allele frequencies were computed using STRAF Software and displayed in Table S2. The number of distinct alleles at each locus varies from 3 for DYS437 to 12 for DYS518 and DYS570. Using the same tool, values for Gene Diversity (GD), Polymorphism Information Content (PIC), Match Probability (PM), and Power of Discrimination (PD) were also determined. The results are shown in Table S3, where loci DYS627 and DYS449 had the highest GD (GD = 0.8148 and 0.8118, respectively), while locus DYS393 had the lowest GD (GD = 0.4858). Eight individuals presented inter-alleles, 7 in locus DYS458 and 1 in DYS448. Four people had duplicated alleles, with values of 10, 12 and 10, 11 in locus DYS439; 28, 29 in locus DYS389II and 14, 15 in locus DYS19. Out of these four samples, 3 belong to Haplogroup R, sub-haplogroup R1b, and it wasn’t possible to predict the Haplogroup for the one with duplicated alleles in locus DYS19. For the bi-allelic locus DYS385a/b, 39 samples (15.47%) were found to be mono-allelic. The results showed no off-ladder alleles or null alleles.

### 3.2. Haplogroup Prediction and Network Analysis

**Figure 2:**
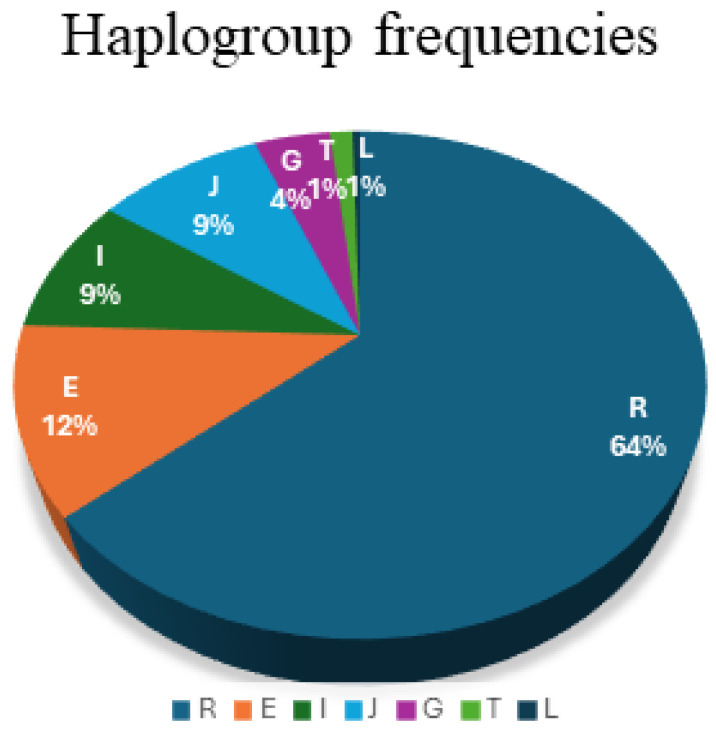
Haplogroup frequencies in the Northern Portuguese population.

Haplogroups were predicted using NevGen Y-DNA Haplogroup Predictor (https://www.nevgen.org/#, accessed on 14 June 2025) with a probability greater than 50%. Haplogroups found include R (64%) and E (12%), as well as other less common ones such as I (9%), J (9%), G (4%), L (1%) and T (1%). The most common sub-haplogroups were R1b (64%), E1b1b (12%), J2a1 (6%) and I1 (4%). Table S4 displays the Haplogroup predictions, while Figure 2 displays the Haplogroup distributions. The percentages of Haplogroups predicted are shown in Table 1.

### 3.3. Population Comparison Using Genetic Distances Based on R_ST_ Distances

The Northern Portuguese population’s haplotypes were compared with those of six other populations—Spain, Italy, Greece, Morocco, Angola and Mozambique, retrieved from YHRD databases, and additionally with two distinct populations from earlier Portuguese population studies, one from the Central region and one from the Azores. Both of these comparisons were used to explore the genetic relationships between the Northern Portuguese population and other South European countries, as well as North African countries and other Portuguese populations.

Because there was insufficient information available for the other loci in the populations that were part of the first comparative analysis, only 17 STR loci were taken into consideration. DYS389I, DYS391, DYS389II, DYS390, DYS458, DYS393, DYS19, DYS437, YGATAH4, DYS448, DYS439, DYS438, DYS456, DYS635, DYS385 a/b and DYS392 were the 17 STRs used.

For the second comparative analysis, where the three Portuguese populations were utilised, only 9 STR loci were taken into consideration for the same reason as above, with those 9 being DYS389I, DYS391, DYS389II, DYS390, DYS393, DYS19, DYS385 a/b and DYS392. The population pairwise genetic distances (**R**_ST_) between the population under study and the previously published populations were computed, presented in Tables S5 and S6, and Central Portugal (**R**_ST_ = −0.00237, *p*-value~0.86486), Spain (**R**_ST_ = −0.00201, *p*-value~0.81982) and Italy (**R**_ST_ = −0.00159, *p*-value~0.35135) were the populations with the most genetic similarity with Northern Portugal. The Rst matrix’s *p*-values revealed a statistically significant genetic difference between Morocco (*p*~0.018), Greece (*p*~0.009), Angola (*p*~0.027), Mozambique (*p*~0.009) and the Azores (*p*~0.000), when compared with Northern Portugal.

**Figure 3:**
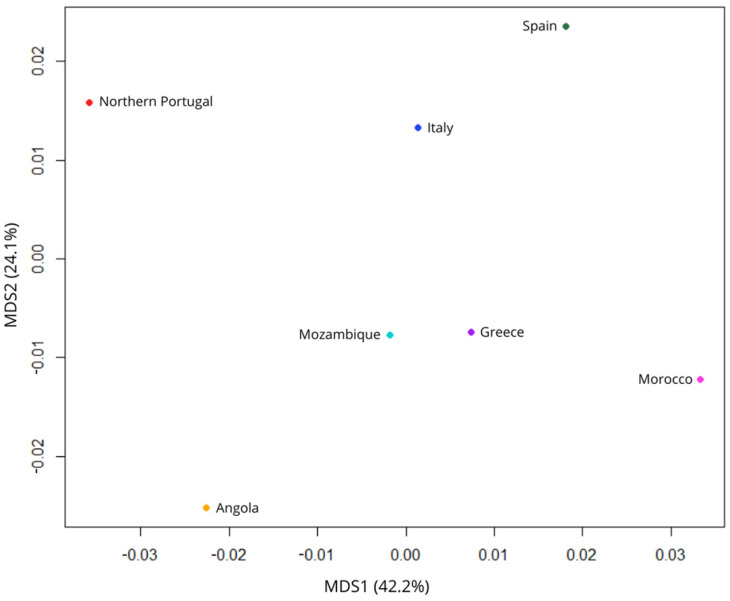
Multidimensional Scaling (MDS) plot based on R_ST_ distances between Northern Portugal and 6 other reference populations using 17 Y-STR data. The plot illustrates genetic similarities between Northern Portugal and European and North African populations. Northern Portugal is closer to Italy and Spain, whilst further away from Angola. The percentages on the axes correspond to the PVE by each dimension.

Figure 3 displays the findings of the MDS plot created to compare the 6 populations. The map indicates that while Greece and Morocco are farther distant from Northern Portugal, Spain and Italy, they are not as far away as Angola and Mozambique. For this set of populations, we computed the Proportion of Variance Explained (PVE), which came out to be 0.663. Additionally, we determined the Krustal stress, 0.62.

To enhance the representability of the data acquired, a 3D MDS plot was computed, because, as we can see, the PVE was relatively high, but the Krustal stress was also significant. This 3D plot can be accessed through the following link: https://drive.google.com/drive/folders/15EqQQT5k9qjA6-6QVAlzwrwlVriVm2Ro?usp=drive_link.

## 4. Discussion

High genetic diversity was found in the 252 Northern Portuguese individuals who were analysed. The high level of genetic variety in the sample is reflected in the frequency of unique haplotypes, which is 0.984 of the 252 haplotypes, of which 251 are distinct and 248 are unique. The discriminating capacity (DC) of 0.992 and high haplotype diversity (HD) of 0.999 were also obtained, confirming the set of markers’ ability to differentiate between people. We can conclude that the panel used in the present work has a higher discriminatory power, as evidenced by comparing these results with those of another Portuguese population study [6]. This may be because there were more Y-STRs present in this study, as we were able to obtain a significantly lower number of shared haplotypes with a very similar number of individuals typed. Whereas Alves et al., 2007 [6] obtained one shared haplotype between three individuals and seventeen haplotypes shared by two individuals, we only found two haplotypes shared by two individuals each. In line with earlier Iberian research like [21], DYS393 displayed the lowest gene diversity among the loci examined, while DYS627 and DYS449 displayed the highest gene diversity, indicating their significance for demographic and forensic investigations. The literature has previously reported the presence of inter-alleles in a Portuguese population, primarily at DYS458 [6]. The Y-chromosome’s mutational processes are also reflected in the detected duplicated alleles, which were previously reported in European populations [22].

Haplogroups for 234 samples could be inferred, and the results of this analysis revealed that the most prevalent haplogroup in the sample pool was R (65%), which is quite frequent in Western Europe, followed by Eb1b (12%), which is common in Iberia despite originating in Africa [23]. R1b was the most prevalent subclade (58.73%), and the Portuguese population’s genetic continuity with other Western European populations is supported by this large R1b presence, which was probably influenced by post-Ice Age recolonisation and subsequent Bronze Age expansions [24]. Previously known as E3b [25], E1b1b is a sub-clade of the E haplogroup, and migrations from the Eastern Mediterranean and North Africa are usually linked to it [26]. It is important to note that haplogroup inference in this study is based on Y-STR and the NevGen predictor. STR-based assignments are uncertain and may lead to misclassification. The historical interpretations presented here should be regarded as broad population-level tendencies consistent with prior studies, rather than definitive phylogenetic conclusions, and are presented only in the context of well-established Iberian paternal lineages. While this approach is informative at a broad population level, STR-based inference does not replace SNP-defined haplogroup determination. Future studies incorporating Y-SNP typing will be necessary to refine phylogenetic resolution and strengthen historical inference.

Populations from Spain [27], Italy [28], Greece [29], Morocco [30], and Angola and Mozambique [31] were included for the comparative analysis. A mix of historical links, sociocultural ties, and geographical proximity supports this choice. Southern European nations include Portugal, Spain, Italy and Greece. Morocco is Portugal’s neighbour in Africa, and the two countries have long-standing cultural and economic ties. Former Portuguese colonies, Angola and Mozambique, have close linguistic, historical and migratory ties to Portugal. The populations of Northern Portugal, Spain, and Italy had little to no significant genetic divergence, according to the results, which is consistent with their close proximity and long-standing Mediterranean ties. However, statistically significant differences were found with Angola, Mozambique, Greece and Morocco, which all had different migratory histories [32,33,34]. The Krustal stress value (0.62) showed some distortion despite the relatively high PVE (66.3%), which was largely addressed in the 3D MDS representation, better honouring the associated *p*-values and R_ST_ values. Central Portugal [35] and the Azores [7] were also compared to Northern Portugal, and while the Azorean population showed significant differentiation from the Northern population, the Northern population showed no statistically significant differences with the Central population. According to Santos et al., 2009 [36], this could be explained by the founder effects and the archipelago’s geographic isolation.

Our results demonstrate a high haplotype diversity and are largely consistent with previous studies on both regional [5] and national [6] Portuguese populations. Minor differences were observed, such as a higher number of rare and duplicated alleles and slightly lower Rst values, which can be explained by the larger sample size and the greater number of Y-STR markers used in this study, confirming the expected genetic variability of the Northern Portugal population. In conclusion, this work reflects the Northern Portuguese male population’s ability to exhibit a high Y-STR haplotype diversity and strong forensic potential. These results also highlight the complex historical influences that shape the region and help to better understand Portugal’s genetic structure.

It is also important to recognize some of the current study’s shortcomings. First, the study’s sample size was limited to 252 males. This figure might not fully reflect the region’s geographic variety. Increasing the amount of sample types could address this, but it might also result in more recurrent haplotypes. Additionally, the frequencies of rare haplotypes, such as T and L, should be taken cautiously because they were based on a very small number of individuals, 3 and 1, respectively. When bigger or other population subgroups are examined, the prevalence of these uncommon lineages may vary significantly. Future research should use larger datasets and supplement Y-STR data with Y-SNP typing, which offers a higher accuracy for haplotype assignment, as we can only predict haplogroups based on Y-STRs and not fully assign them. The limited number of markers used for population comparison should also be addressed, resulting from a lack of information for the reference populations. Including these populations in future studies with a more comprehensive marker set would allow for more accurate and balanced comparisons, thereby reducing potential biases in the results.

## 5. Conclusions

The present study delivers a comprehensive 26-locus Y-STR reference dataset for the Northern Portuguese population, demonstrating a high haplotype diversity, a large proportion of unique haplotypes, and strong discriminatory power, confirming its suitability for forensic applications such as paternity testing, kinship analysis, and criminal investigations. The Y-STR profiles obtained reflect the genetic continuity expected for Western Iberian populations, while also capturing the subtle contributions of Mediterranean and North African lineages, which are consistent with Portugal’s well-documented demographic history. Although haplogroup predictions based solely on STR must be interpreted cautiously, they nonetheless align with known paternal genetic patterns of the Iberian Peninsula.

By contributing these data to the YHRD database, the present work substantially improves the representation of Portuguese populations in international reference resources, enhancing the accuracy and reliability of comparative forensic analysis. Future efforts should focus on expanding sampling to other regions of mainland Portugal, as well as the archipelagos, and integrating Y-SNP data to achieve refined phylogenetic resolution. Together, these steps will support a more complete reconstruction of Portuguese Paternal genetic structure and further consolidate the forensic value of Y-chromosome markers at both national and international levels.

## Figures and Tables

**Table 1:** Haplogroup frequencies in the Northern Portuguese population sample. N represents the number of samples in each sub-haplogroup.

Haplogroup (H)	Sub-Haplogroup (SH)	N (SH)	Frequency SH
R (64%)	R1b	148	0.6435
	R1a	1	0.0043
E (12%)	E1b1b	28	0.1217
I (9%)	I1	9	0.0391
	I2a1a	4	0.0174
	I2a2a	8	0.0348
J (9%)	J1a2a2	2	0.0087
	J1a2a1a2	5	0.0217
	J2a1	13	0.0565
	J2b2a	2	0.0087
G (4%)	G2a2	2	0.0087
	G2a2a	1	0.0043
	G2a2b1	3	0.0130
	G2a2b2a1b	1	0.0043
	G2a2b2a1c	2	0.0087
L (1%)	L1b	1	0.0043
T (1%)	-	-	-

## Data Availability

The original contributions presented in this study are included in this article/Supplementary Materials. Further inquiries can be directed to the corresponding author.

## Supplementary Materials

The following supporting information can be downloaded at https://www.mdpi.com/article/10.3390/genes17010101/s1. Table S1. Haplotype and respective frequency of 252 individuals of the Northern Portuguese Population. Table S2. Haplotypic frequencies for 252 male individuals from Northern Portugal population. Calculated using STRAF. Table S3. Forensic Parameters for the 252 individuals sampled from the Northern Portuguese Population. Calculated using STRAF. Table S4. Haplogroup and sub-haplogroup prediction for 252 individuals from the Northern Portuguese Population. Predicted using NevGen. Table S5. Rst genetic distances and corresponding *p*-values between Northern Portugal and six other reference populations. Table S6. Rst genetic distances and corresponding *p*-values between Northern Portugal and two other Portuguese reference populations. Figure S1. Distribution of 23 Y-STRs Allele frequencies calculated using STRAF.

## Abbreviations

The following abbreviations are used in this manuscript:

DC	Discriminatory Capacity
DNA	Deoxyribonucleic Acid
GD	Gene Diversity
HD	Haplotype Diversity
MDS	Multidimensional scaling
MP	Match Probability
PCR	Polymerase chain reaction
PD	Power Discrimination
PIC	Polymorphism Information Content
PVE	Proportion of Variance Explained
RM-STRs	Rapid Mutation Short Tandem Repeats
STR	Short Tandem Repeats
UHF	Unique Haplotype Frequency
YHRD	Y-chromosome Haplotype Reference Database
Y-STRs	Y-chromosome short tandem repeats

## References

[1] Campos  D.R., Ribeiro F., Guerreiro J., Gabriel L., Miranda R., Machado S. Norte. Onde Começa Portugal. História. Projeto Norte 2014. https://projetonorte.wordpress.com/norte/historia/.

[2] Jordana X., Malgosa A., Casté B., Tornero C. (2019). Lost in transition: The dietary shifts from Late Antiquity to the Early Middle Ages in the North Eastern Iberian Peninsula. Archaeol. Anthropol. Sci..

[3] Curchin L. (2014). Roman Spain (Routledge Revivals): Conquest and Assimilation.

[4] Mattos J. (1988). Identificação de um País: Oposição-Composição: Ensaio Sobre as Origens de Portugal: 1096-1325.

[5] Pontes M.L., Cainé L., Abrantes D., Lima G., Pinheiro M.F. (2007). Allele frequencies and population data for 17 Y-STR loci (AmpFlSTR Y-filer) in a Northern Portuguese population sample. Forensic Sci. Int..

[6] Alves C., Gomes V., Prata M.J., Amorim A., Gusmão L. (2007). Population data for Y-chromosome haplotypes defined by 17 STRs (AmpFlSTR YFiler) in Portugal. Forensic Sci. Int..

[7] Carvalho M., Anjos M.J., Andrade L., Lopes V., Santos M.V., Gamero J.J., Corte Real F., Vide M.C. (2003). Y-chromosome STR haplotypes in two population samples: Azores Islands and Central Portugal. Forensic Sci. Int..

[8] Quintana-Murci L., Fellous M. (2001). The Human Y Chromosome: The Biological Role of a “Functional Wasteland”. J. Biomed. Biotechnol..

[9] Sánchez-Méndez A.D., Narvaez-Rivera S.E., Rangel-Villalobos H., Hernández-Bello J., López-Quintero A., Moreno-Ortíz J.M., Ramos-González B., Aguilar-Velázquez J.A. (2025). Genetic Diversity and Forensic Parameters of 27 Y-STRs in Two Mestizo Populations from Western Mexico. Genes.

[10] Syndercombe Court D. (2021). The Y chromosome and its use in forensic DNA analysis. Emerg. Top. Life Sci..

[11] Bonito M., D’Atanasio E., Ravasini F., Cariati S., Finocchio A., Novelletto A., Trombetta B., Cruciani F. (2021). New insights into the evolution of human Y chromosome palindromes through mutation and gene conversion. Hum. Mol. Genet..

[12] Tanudisastro H.A., Deveson I.W., Dashnow H., MacArthur D.G. (2024). Sequencing and characterizing short tandem repeats in the human genome. Nat. Rev. Genet..

[13] Parkin E.J., Kraayenbrink T., van Driem G.L., Tshering Of Gaselô K., de Knijff P., Jobling M.A. (2006). 26-Locus Y-STR typing in a Bhutanese population sample. Forensic Sci. Int..

[14] Willuweit S., Roewer L. (2007). Y chromosome haplotype reference database (YHRD): Update. Forensic Sci. Int. Genet..

[15] Biosystems A. (2017). PrepFiler Express™ and PrepFiler Express BTA™ Forensic DNA Extraction Kits. https://assets.thermofisher.com/TFS-Assets/LSG/manuals/cms_081933.pdf.

[16] Qiagen (2022). Investigator^®^ Argus Y-28 QS Handbook. https://www.qiagen.com/us/resources/resourcedetail?id=cf7ae42a-72bc-4d3d-812d-684d0625a179〈=en.

[17] Nei M., Tajima F. (1981). Genetic drift and estimation of effective population size. Genetics.

[18] Gouy A., Zieger M. (2017). STRAF-A convenient online tool for STR data evaluation in forensic genetics. Forensic Sci. Int. Genet..

[19] Excoffier L., Laval G., Schneider S. (2007). Arlequin (version 3.0): An integrated software package for population genetics data analysis. Evol. Bioinform. Online.

[20] Team R.C. (2024). R: A Language and Environment for Statistical Computing. https://www.r-project.org.

[21] Gaibar M., Esteban E., Moral P., Gómez-Gallego F., Santiago C., Bandrés F., Luna F., Fernández-Santander A. (2010). STR genetic diversity in a Mediterranean population from the south of the Iberian Peninsula. Ann. Hum. Biol..

[22] Schmidt U., Meier N., Lutz S. (2003). Y-chromosomal STR haplotypes in a population sample from southwest Germany (Freiburg area). Int. J. Leg. Med..

[23] Flores C., Maca-Meyer N., González A.M., Oefner P.J., Shen P., Pérez J.A., Rojas A., Larruga J.M., Underhill P.A. (2004). Reduced genetic structure of the Iberian peninsula revealed by Y-chromosome analysis: Implications for population demography. Eur. J. Hum. Genet..

[24] Olalde I., Mallick S., Patterson N., Rohland N., Villalba-Mouco V., Silva M., Dulias K., Edwards C.J., Gandini F., Pala M. (2019). The genomic history of the Iberian Peninsula over the past 8000 years. Science.

[25] Cruciani F., La Fratta R., Santolamazza P., Sellitto D., Pascone R., Moral P., Watson E., Guida V., Colomb E.B., Zaharova B. (2004). Phylogeographic analysis of haplogroup E3b (E-M215) y chromosomes reveals multiple migratory events within and out of Africa. Am. J. Hum. Genet..

[26] Alvarez L., Santos C., Montiel R., Caeiro B., Baali A., Dugoujona J.M., Aluja M.P. (2009). Y-chromosome variation in South Iberia: Insights into the North African contribution. Am. J. Hum. Biol..

[27] Purps J., Siegert S., Willuweit S., Nagy M., Alves C., Salazar R., Angustia S.M., Santos L.H., Anslinger K., Bayer B. (2014). A global analysis of Y-chromosomal haplotype diversity for 23 STR loci. Forensic Sci. Int. Genet..

[28] Piglionica M., Baldassarra S.L., Giardina E., Stella A., D’Ovidio F.D., Frati P., Lenato G.M., Resta N., Dell’Erba A. (2013). Population data for 17 Y-chromosome STRs in a sample from Apulia (Southern Italy). Forensic Sci. Int. Genet..

[29] Kovatsi L., Saunier J.L., Irwin J.A. (2009). Population genetics of Y-chromosome STRs in a population of Northern Greeks. Forensic Sci. Int. Genet..

[30] Aboukhalid R., Bouabdellah M., Abbassi M., Bentayebi K., Elmzibri M., Squalli D., Amzazi S. (2010). Haplotype frequencies for 17 Y-STR loci (AmpFlSTRY-filer) in a Moroccan population sample. Forensic Sci. Int. Genet..

[31] Palha T., Gusmão L., Ribeiro-Rodrigues E., Guerreiro J.F., Ribeiro-Dos-Santos A., Santos S. (2012). Disclosing the genetic structure of Brazil through analysis of male lineages with highly discriminating haplotypes. PLoS ONE.

[32] Arenas F. (2015). Migrations and the Rise of African Lisbon: Time-Space of Portuguese (Post)coloniality. Postcolon. Stud..

[33] Kalter C. (2024). Building Nations After Empire: Post-Imperial Migrations to Portugal in a Western European Context. Contemp. Eur. Hist..

[34] Van Dommelen P. (2012). Colonialism and migration in the ancient Mediterranean. Annu. Rev. Anthropol..

[35] Bento A.M., Lopes V., Serra A., Costa H.A., Andrade L., Balsa F., Anjos M.J., Carvalho M., Oliveira C., Batista L. (2007). Population data for Y-chromosome haplotypes defined by 17 STRs (AmpFlSTR YFiler) in Central Portugal. Forensic Sci. Int. Genet. Suppl. Ser..

[36] Santos C., Alvarez L., Aluja M.P., Bruges-Armas J., Lima M. (2009). Genetic structure of the Azores Islands: A study using 15 autosomal short tandem repeat loci. Coll. Antropol..

