# Supplementary figures and images for: Population of Northern Portugal: Study of Genetic Diversity and Forensic Parameters of 26 Y-STR Markers
Source: Genes (Basel). 2026 Jan 19;17(1):101. doi: 10.3390/genes17010101 (PMC12841522; doi:10.3390/genes17010101)

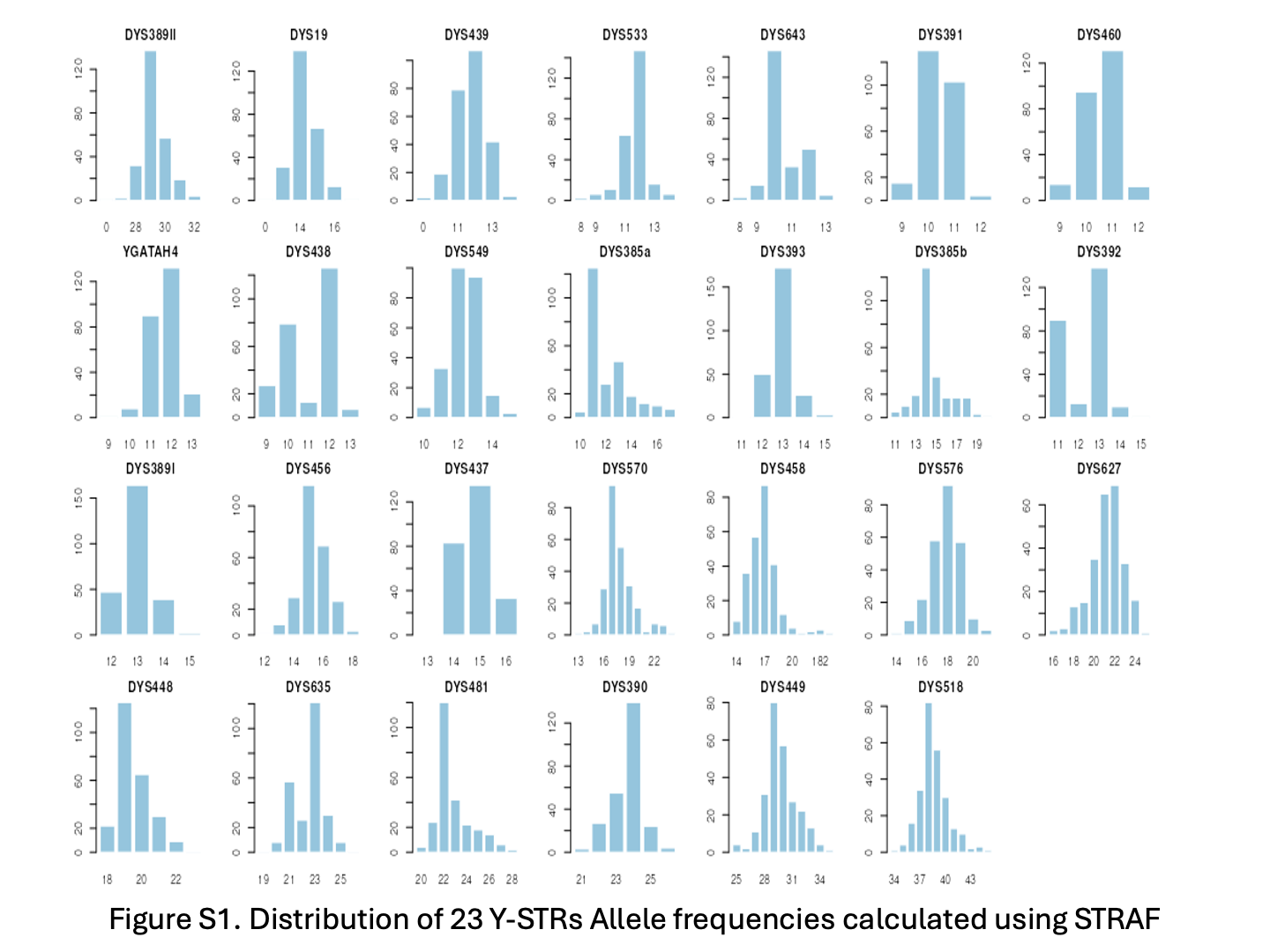

Supplement: Supplementary file 1 [file genes-17-00101-s001.zip › Figure S1.png]
